# Enhancing Asthma Management in Emergency Departments: New Approaches Are Vital for Effective Care

**DOI:** 10.1002/resp.70284

**Published:** 2026-07-06

**Authors:** Christopher C. Price, Mariko S. Koh, Fanny W. Ko, Philip G. Bardin, Paul Leong

**Affiliations:** ^1^ Monash Lung Sleep Allergy & Immunology, Monash Health Clayton Victoria Australia; ^2^ School of Clinical Sciences at Monash Health Clayton Melbourne Victoria Australia; ^3^ Department of Respiratory and Critical Care Medicine Singapore General Hospital Singapore Singapore; ^4^ Duke‐National University of Singapore Medical School Singapore Singapore; ^5^ The Chinese University of Hong Kong Hong Kong China

**Keywords:** asthma, asthma management, beta‐agonist, comprehensive care, corticosteroid, emergency department

1

Approximately 11% of Australians live with asthma whilst Asia‐Pacific countries report a prevalence of 5%–20% [1]. The disease can be fatal and it is still responsible for 250,000 deaths globally every year, many of which are preventable [1]. A well‐known risk factor for poor outcomes, including mortality, is emergency department (ED) attendance [2].

EDs play an important role in asthma care globally, and in Australia, there were more than 60,000 presentations related to asthma in 2024–2025 [3]. EDs are an important pillar of asthma management in the Asia‐Pacific, with up to 32% of asthma patients having at least one ED presentation annually [4]. Presentations are highest in regional and remote areas and among those living in socially disadvantaged regions where EDs may be the only form of accessible healthcare [3]. Consequently, for many people, EDs are the dominant way in which they receive asthma care, and this reliance is likely to be greatest in communities that face systemic barriers to care.

Theoretically, each ED presentation is an opportunity to implement ideal asthma care, improve asthma control and potentially avert life‐threatening asthma events. However, recent data examining three key tenets of asthma management in EDs reveal that patterns of current practice fall short of these ambitions.

First, treating asthma with unopposed short acting beta agonists (SABAs) is dangerous, leading to tachyphylaxis and increased risk of death [2]. SABA overuse begets poor symptom control and a greater risk of asthma exacerbation [2]. GINA 2026 does not recommend SABA monotherapy at any step of asthma treatment [2]. Recent Australian data show that approximately 50% of patients with a known history of asthma presenting to ED were on SABA‐only therapy, representing a significant opportunity for ICS initiation [5, 6]. On discharge from the ED, however, 35%–41% of patients remain on SABA monotherapy [5], exposing them to significant risk.

Second, inhaled corticosteroids (ICS) are a mainstay of asthma management and all global guidelines emphasise their critical role in reduction of asthma harm including exacerbations and death. However, Australian data in four EDs showed that only 7%–23% of patients presenting with an asthma exacerbation to ED who were ICS naïve were discharged on ICS therapy [5, 6, 7, 8]. ICS discharge description is consistently poor across the Asia Pacific region (Figure 1a). In Hong Kong, only 65% of patients reviewed in ED were on some form of ICS containing therapy and among those presenting two or more times to ED, 17% were not initiated on ICS [10]. Similarly, 16% of patients used SABA alone and, of greater concern, 17% of patients did not use any inhaled asthma medication [10]. In Singapore, poor rates of guideline compliance were found and ICS were prescribed in 27% of patients at ED discharge [9].

**FIGURE 1:**
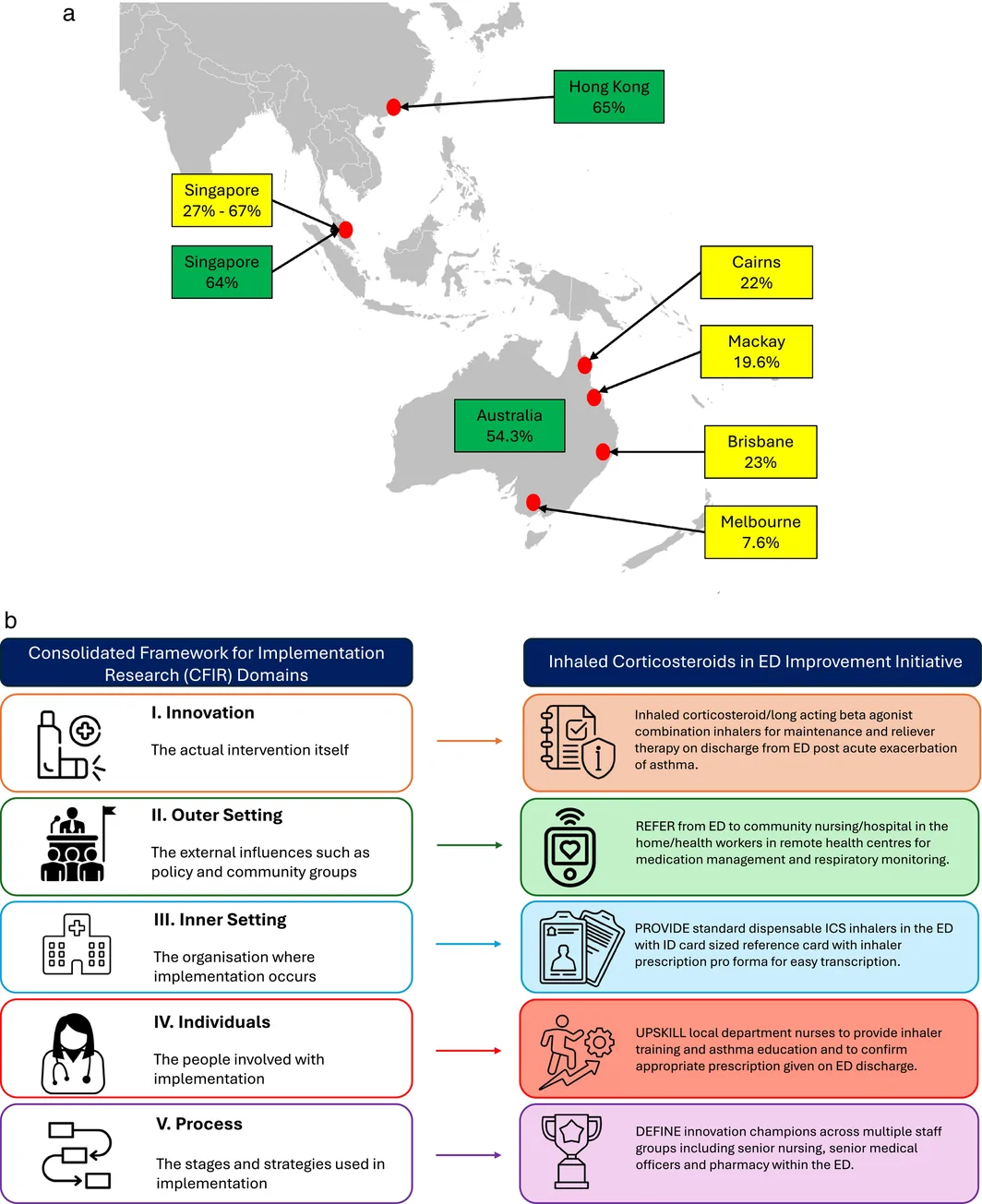
(a) Rates of inhaled corticosteroid prescription at discharge from Emergency Departments after admission for an acute asthma exacerbation in patients not already on inhaled corticosteroids (yellow box) [5, 6, 7, 8, 9]. Rates of inhaled corticosteroid prescription on presentation to Emergency Departments (green box) [9, 10, 11]. (b) Hypothetical example intervention to increase ED ICS usage using the Consolidated Framework for Implementation Research (CFIR) [12].

The reasons for the geographical variation in ICS prescription are not well established and require research. It is highly likely that ICS prescription rates exist at the intersection of department culture, clarity of recommendations, health care skillsets and resourcing with varying facilitators and barriers. However, it is clear that lifting ICS prescription rates should be a key focus of any ED intervention.

Third, patient education and self‐management are of prime importance. ED interventions aimed at education and inhaler management can improve patient knowledge, reduce the risk of unscheduled ED visits and improve quality of life [13]. However, the use of asthma action plans in Australian EDs on discharge was approximately 10% to 23% [5]. Hong Kong EDs demonstrated a similarly low rate of action plan usage at 10% [10]. In one Singapore ED, only half of patients received any form of asthma education [14].

These reports speak to a crucial gap in implementation of recommended asthma care. The evidence and the guidelines exist, but ED practice is not yet aligned. Why is this the case? Several important impediments exist. Asthma management has rapidly changed over the past decade and knowledge dissemination to ED and other providers is an ongoing challenge. Staffing shortfalls, the need to ensure that high acuity patients are stabilised, and increasing demands on ED workflows mean that there is considerable time pressure on the provision of asthma care in the ED [15]. Conceptually there is also uncertainty about the role of ED staff in preventative and long‐term care [15].

What solutions exist? A Singapore hospital introduced an Asthma‐COPD after‐hours Respiratory Nurse at Emergency (A‐CARE) providing after‐hours nursing support in ED [9]. The nurse delivered disease education, inhaler technique training and assessment of pharmacological interventions and facilitated significant improvements in OCS prescriptions, ICS initiation and follow up planning over 17 months. Importantly, prior to developing this intervention, local barriers to guideline implementation were researched through a quality improvement project. Several important factors were identified including availability of relevant equipment, lack of knowledge about guidelines and after‐hours limitations. Positive facilitators include making interventions modest and simple, ensuring adequate training and resourcing, streamlining pathways to general practice as well as funding resources to provide education and inhaler technique training.

More generally, Implementation Science offers a flexible ‘roadmap’ that can be used to develop and scale interventions to improve ED asthma care, manage change, and ultimately optimise asthma management. Implementation Science Models including the Consolidated Framework for Implementation Research (CFIR) and RE‐AIM allow a rigorous, thorough examination of factors that drive or hinder optimal implementation, in addition to measurement and evaluation of outcomes [12]. They have been successfully used across a wide variety of healthcare interventions and could be used to improve asthma care [12]. Figure 1b showcases an illustrative example of a CFIR approach to improve inhaled corticosteroid prescription in ED. Research is needed to evaluate how implementation science can be operationalised, evaluated and implemented at scale in EDs to improve asthma care.

In summary, improving asthma care in EDs across the Asia‐Pacific represents a major opportunity to enhance asthma control for a population at the highest risk of poor asthma outcomes. Changing asthma care in ED will require a coordinated, concerted multidisciplinary effort underpinned by rigorous science.

## Author Contributions

**Paul Leong:** supervision, writing – review and editing, conceptualization. **Fanny W. Ko:** writing – review and editing, data curation, resources. **Philip G. Bardin:** supervision, writing – review and editing, conceptualization. **Christopher C. Price:** writing – original draft, writing – review and editing, conceptualization, investigation. **Mariko S. Koh:** writing – review and editing, data curation, resources.

## Funding

C.C.P. is supported by the Monash Lung and Sleep Institute (PhD scholarship). The funder had no influence on the preparation of this manuscript.

## Conflicts of Interest

P.L. declares travel support from GSK, Sanofi. M.S.K. and F.W.K. are Editorial Board members of *Respirology* and were excluded from all editorial decision‐making related to this article.

## Data Availability

The data that support the findings of this study are available from the corresponding author upon reasonable request.
